# A molecular mechanism realizing sequence-specific recognition of nucleic acids by TDP-43

**DOI:** 10.1038/srep20576

**Published:** 2016-02-03

**Authors:** Yoshiaki Furukawa, Yoh Suzuki, Mami Fukuoka, Kenichi Nagasawa, Kenta Nakagome, Hideaki Shimizu, Atsushi Mukaiyama, Shuji Akiyama

**Affiliations:** 1 Laboratory for Mechanistic Chemistry of Biomolecules, Department of Chemistry, Keio University, Yokohama 223-8522, JAPAN; 2 RIKEN Center for Life Science Technologies, Yokohama 230-0045, JAPAN; 3 Research Center of Integrative Molecular Systems (CIMoS), Institute for Molecular Science, Okazaki 444-8585, JAPAN; 4 Department of Functional Molecular Science, SOKENDAI (The Graduate University for Advanced Studies), Okazaki 444-8585, JAPAN

## Abstract

TAR DNA-binding protein 43 (TDP-43) is a DNA/RNA-binding protein containing two consecutive RNA recognition motifs (RRM1 and RRM2) in tandem. Functional abnormality of TDP-43 has been proposed to cause neurodegeneration, but it remains obscure how the physiological functions of this protein are regulated. Here, we show distinct roles of RRM1 and RRM2 in the sequence-specific substrate recognition of TDP-43. RRM1 was found to bind a wide spectrum of ssDNA sequences, while no binding was observed between RRM2 and ssDNA. When two RRMs are fused in tandem as in native TDP-43, the fused construct almost exclusively binds ssDNA with a TG-repeat sequence. In contrast, such sequence-specificity was not observed in a simple mixture of RRM1 and RRM2. We thus propose that the spatial arrangement of multiple RRMs in DNA/RNA binding proteins provides steric effects on the substrate-binding site and thereby controls the specificity of its substrate nucleotide sequences.

TAR DNA-binding protein-43 (TDP-43) is a DNA/RNA-binding protein, which has multiple physiological functions such as alternative splicing, miRNA processing, and mRNA turnover^1^. The lethality of the conventional knockout^2^,^3^ and the post-natal deletion of TDP-43 in mice^4^ highlights the essential physiological roles of TDP-43. In 2006, furthermore, TDP-43 was found as an aggregated protein consisting of abnormal inclusions in several neurodegenerative diseases such as frontotemporal lobar degeneration (FTLD) and amyotrophic lateral sclerosis (ALS)^5^,^6^, and later, mutations in the TDP-43 gene were identified as a cause of familial forms of FTLD and ALS^7^,^8^,^9^,^10^. Since then, roles of TDP-43 in the pathomechanism of these diseases have been intensively studied; however, it remains controversial whether the cause of neurodegeneration is linked to the gain of pathogenic toxicity or the loss of the physiological functions of TDP-43. Hence, a molecular mechanism regulating the recognition and substrate-binding of TDP-43 to RNA/DNA will significantly help us understand the various pathological as well as physiological processes involved.

TDP-43 is known to selectively bind UG-repeated/enriched RNA and TG-repeated single-stranded DNA (ssDNA) at its two RNA-recognition motifs (RRM1 and RRM2); indeed, one of the physiological functions of TDP-43 is to bind UG-repeats near the splicing site of pre-mRNA transcripts and promote exon skipping or inclusion^11^,^12^. Typically, RRM is composed of *approx.* 90 amino acids that fold into a β1α1β2β3α2β4 topology with highly conserved segments (6–8 a.a.) in β1 and β3 (called RNP2 and RNP1, respectively)^13^. Aromatic and positively charged residues in RNP1/2 play crucial roles in the binding of RNA/ssDNA and are conserved in both RRM1 and RRM2 of TDP-43^11^. In fact, amino acid mutations in RNP1 of RRM1 resulted in a decreased affinity of TDP-43 to RNA/ssDNA; in contrast, the affinity to RNA/ssDNA was not affected with mutations in the corresponding site in RRM2^14^. This is enigmatic because RRM2 appears to fulfill structural requirements for the binding of RNA/ssDNA. While it is evident that RRM1 is responsible for binding RNA/ssDNA, the role of RRM2 in the functions of TDP-43 is quite obscure.

There are several precedents of RRMs that do not directly bind RNA/DNA^13^. RRM, which is known as one of the most abundant domains in the human proteome^15^, often exists as multiple copies in a single protein; thereby allowing for sophisticated functions that otherwise could not be performed with a single RRM^16^. In TDP-43, RRM2 follows RRM1 in tandem *via* a 14-aa linker; therefore, the interplay between RRM1 and RRM2 would be essential to the physiological functions of TDP-43. Actually, based upon the NMR structure of RRM1-RRM2 (RRM1–2) complexed with UG-enriched RNA, RRM2 is proposed to have roles in aligning the bound RNA in a specific direction for successful splicing in cooperation with other splicing factors^17^. In the RRM1–2 and UG-enriched RNA complex, furthermore, stacking/hydrophobic interactions have been described between the phenylalanine residues (F194, F229, F231) in RNP1/2 of RRM2 and U/G nucleotides^17^, but it is also notable that mutations at RNP1 (F229A/F231A) of RRM2 reduces the binding affinity by only two-fold for UG-repeated RNA^18^. Further investigation is hence definitely required to explain why TDP-43 possesses two sets of RRMs.

To elucidate the significance of RRM multiplicity in the RNA/ssDNA recognition by TDP-43, we have systematically examined the binding of RRM1, RRM2, and RRM1–2 with ssDNA containing an NN′-repeated sequence, where N and N′ represent either A, T, G, or C. Contribution of interplay between RRM1 and RRM2 to the binding of specific sequences was also probed by introducing mutations at the interface between RRM1 and RRM2. So far, fusion of RRM2 with RRM1 has been shown to increase the affinity to TG/UG-rich ssDNA/RNA through the nucleotide-specific interactions in RRM2^17^. Here, we are proposing a new mechanism that regulates the sequence-specific recognition by multiple RRMs. Namely, RRM1 is equipped with several distinct binding sites for RNA/ssDNA, but RRM2 occludes some of those sites in RRM1. Such steric hindrance of RRM2 is thus considered to contribute to the specific binding of TG/UG-rich nucleotides by RRM1–2. We thus suggest that the spatial arrangement of RRM1 and RRM2 in TDP-43 determines sequence-specificity upon the binding of RNA/ssDNA and that any disturbance to the spatial arrangement of RRMs would retard some of the physiological functions of TDP-43.

## Results and Discussion

### RRM1 but not RRM2 has the ability to bind RNA

**Figure 1:**
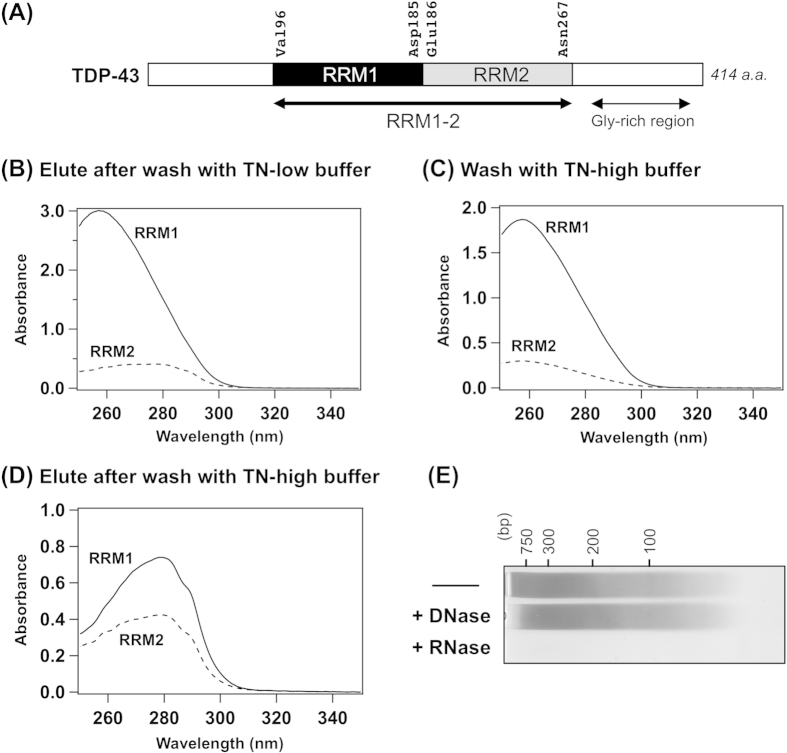
RRM1 but not RRM2 is co-purified with endogenous RNA from *E. coli* lysates. **(A)** Schematic representation of a TDP-43 domain structure. **(B**–**D)** RRM1 (solid curve) and RRM2 (broken curve) were overexpressed in *E. coli* BL21(DE3) and purified with Ni^2+^-affinity chromatography. (**B**) Crude lysates were loaded on the Ni^2+^-affinity resins, which were washed with a TN-low buffer. The bound proteins were eluted from the resins and examined spectroscopically. (**C**,**D**) Resins incubated with crude lysates were washed with a TN-high buffer, and the bound proteins were eluted. Fractions obtained in (**C**) the wash and (**D**) the elute steps were examined spectroscopically. **(E)** The fraction washed out by a TN-high buffer from the resins incubated with RRM1 crude lysates was treated with either DNase or RNase and analyzed with urea-PAGE.

To evaluate the RNA binding ability of RRMs in TDP-43 *in vitro*, recombinant RRM1 and RRM2 (Fig. 1A) with an N-terminal His tag were overexpressed in *E. coli* and purified with Ni^2+^-affinity resins. Notably, at the final step of purification using a buffer containing 100 mM NaCl (a TN-low buffer, see experimental procedures), an absorption band was observed at 260 nm in the purified RRM1 sample but not RRM2, showing that RRM1 was co-purified with endogenous DNA/RNA of *E. coli* (Fig. 1B). When the resins mixed with crude RRM1 sample were washed with a buffer containing 1 M NaCl (a TN-high buffer, see experimental procedures), endogenous DNA/RNA showing absorption at 260 nm were washed out (Fig. 1C), and RRM1 proteins free from DNA/RNA were obtained (Fig. 1D). The species washed out from the resins by a TN-high buffer were furthermore digested with RNase but not DNase (Fig. 1E); therefore, these results show that RRM1 but not RRM2 can bind endogenous RNA in *E. coli*.

**Figure 2:**
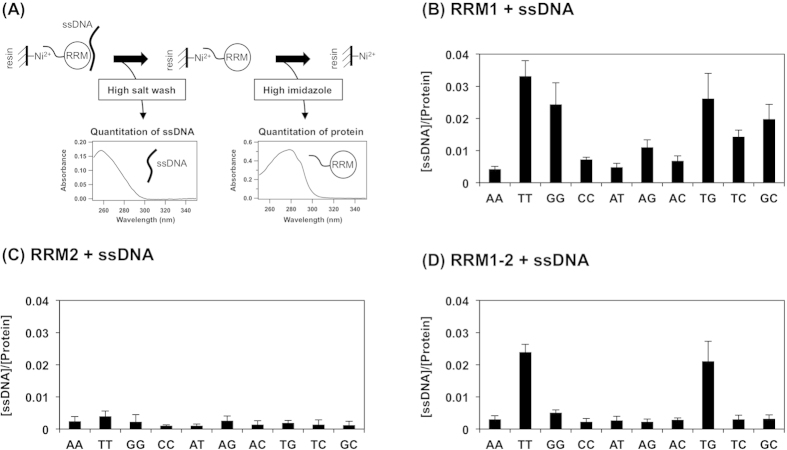
RRM proteins exhibit distinct binding abilities of ssDNA in a sequence-specific manner. (**A)** A schematic representation of a pull-down assay using His-tagged RRM proteins and Ni^2+^-affinity resins. **(B**–**D)** The molar ratio between ssDNA and RRM proteins (RRM1, RRM2 and RRM1–2 in (**B**–**D**), respectively) in a pull-down assay was plotted against NN′, which describes a repeating part in an ssDNA sequence, 5′-A_10_(NN′)_5_A_10_-3′. An exception is TT, which is an ssDNA with the sequence, 5′-C_10_(TT)_5_C_10_-3′. More than three independent experiments were performed to estimate error bars (standard deviations).

Distinct binding affinities (*K*_d_) of RRM1 and RRM2 to (U/T)G repeats have been estimated in the nanomolar and micromolar range, respectively^18^,^19^,^20^. Based upon the structure of an RRM1/ssDNA complex, the possibility that RRM1 could bind non-(U/T)G repeats have been described^18^. Despite this, little experimental information has been reported on their affinities to nucleotides with sequences other than (U/T)G repeats. To test the affinity of RRM1 and RRM2 to the nucleotides with various sequences, therefore, we have developed a pull-down assay by using ssDNA. While RNA is generally prone to chemical as well as enzymatic degradation, ssDNA has higher stability for easier handling. Besides, ssDNA has often been used as a model of RNA for the examination of the binding affinity of TDP-43 toward nucleic acids^14^. In our pull-down assay (Fig. 2A), the Ni^2+^-affinity resins were first charged with purified His-tagged RRM proteins and then incubated with ssDNA. Ten sequences of ssDNA, 30 nucleotides in length, were examined for the assay: 5′-A_10_(NN′)_5_A_10_-3′(NN′: AA, AT, AG, AC, TG, TC, GG, GC, or CC) and 5′-C_10_(TT)_5_C_10_-3′. Because RRMs in TDP-43 have been shown to accommodate nine or ten nucleotides upon interaction with ssDNA/RNA^17^,^18^, the ssDNAs modeled in this study have sufficient numbers of nucleotides in length for examination of their interactions with RRMs. Furthermore, the binding affinity of TDP-43 RRMs with (T/U)G-repeated nucleotides is known to depend upon its repeat number^11^,^14^,^19^, and RNA with five but not three repeats of UG has been reported to form a significantly tight complex with TDP-43 (nanomolar *K*_d_)^11^. After washing out unbound ssDNA with TN-low buffer, the ssDNA bound to RRM proteins was eluted with TN-high buffer. The RRM proteins bound to the resins were finally eluted with imidazole. Judging from the spectral shape of the eluted samples, little cross-contamination of ssDNA and RRM proteins was confirmed, which allowed us to quantify ssDNA and RRM proteins based upon the absorption at 260 and 280 nm, respectively (Fig. 2A). Among all the samples tested, equal amounts (~3 nmol) of RRM proteins were bound to the resins; however, ssDNAs were detected in RRM1 samples but not in RRM2 (Fig. 2B,C). Namely, in addition to TG repeats, RRM1 is found to bind ssDNA with other sequences (Fig. 2B). A weak affinity of RRM2 to TG repeats was reproduced, but we have found that other sequences were also not well recognized by RRM2 (Fig. 2C). While the inability of RRM2 to bind ssDNA might indicate possible misfolding of RRM2, circular dichroism (CD) analysis has supported the folding of both RRM1 and RRM2 prepared in this study, which will be described later in more detail. Although RRM2 may bind ssDNA/RNA with specific sequences that were not tested in this study^21^, these results support RRM1 as having the primary role in the ssDNA/RNA binding of native TDP-43.

### RRM2 “fine-tunes” the sequence specificity of ssDNA recognized by TDP-43

In TDP-43, RRM1 and RRM2 were placed in close proximity and linked in tandem (RRM1–2). Even though RRM2 alone did not bind ssDNA, RRM2 would modulate the interaction between ssDNA and RRM1. We thus repeated the pull-down assay using RRM1–2, in which RRM1 and RRM2 are fused as in native TDP-43 (Fig. 1A), and found that RRM1–2 exclusively bound ssDNA containing TT- and TG-repeats (Fig. 2D). This is again consistent with previous reports that TDP-43 binds TG-repeated ssDNA and UG-repeated RNA^11^,^12^, while the binding of TT repeats (5′-C_10_(TT)_5_C_10_-3′) with RRM1–2 was unexpected. Our results on TT repeats are not consistent with the study by Mackness *et al*.^20^, in which no detectable interactions between TDP-43 RRMs and T_12_ oligonucleotide were reported. We thus repeated our pull-down assay with T_12_ and T_30_ oligonucleotides, and our RRM1 and RRM1–2 but not RRM2 pulled down those oligonucleotides (data not shown). While the discrepancy in the binding of T-repeats between Mackness *et al*. and ours still remains obscure, we suppose that RRM1–2 can bind nucleotides containing T-repeats. Actually, the interaction of RRM1–2 with T-repeats might be supported by a previous finding in which intracellular TDP-43 can be cross-linked with RNAs containing the sequence, UUUUU^17^,^22^. Nonetheless, we would like to emphasize here that the ssDNA recognition by RRM1–2 had significantly higher sequence-specificity compared to RRM1 alone. For example, RRM1 exhibited significant affinity to GG-repeated as well as TG-repeated ssDNAs (Fig. 2B); in contrast, GG-repeated ssDNA was not pulled down with RRM1–2 (Fig. 2D). Based upon these results, RRM2 appears to have important roles in increasing (or “fine-tuning”) the sequence-specificity of ssDNA recognized by RRM1.

**Figure 3:**
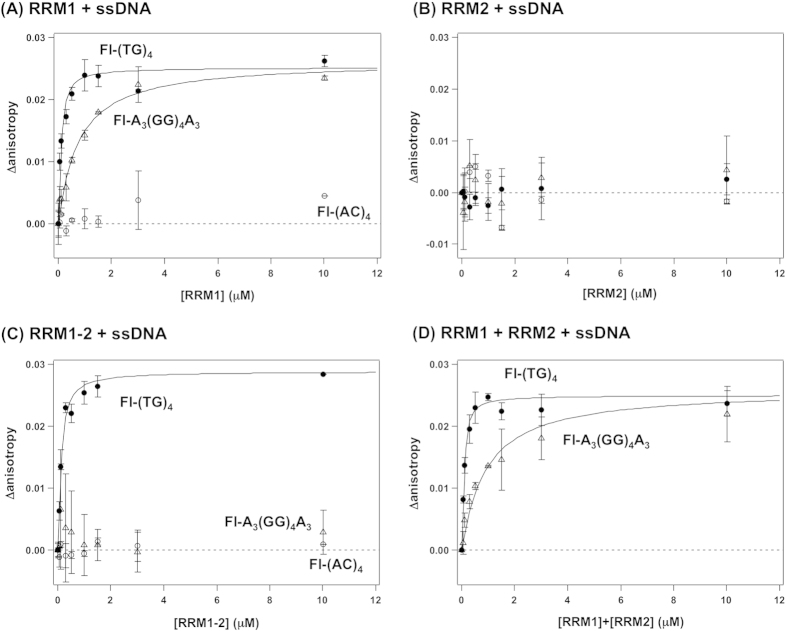
Analysis of the sequence-dependent interaction between RRM proteins and ssDNA by fluorescence anisotropy measurements. 0.1 μM Fl-(TG)_4_ (filled circles), Fl-(AC)_4_ (open circles), and Fl-A_3_(GG)_4_A_3_ (open triangles) were titrated with **(A)** RRM1, **(B)** RRM2 **(C)** RRM1–2 and **(D)** an equimolar mixture of RRM1 and RRM2, and the fluorescence anisotropy was measured. To estimate the dissociation constant, *K*_d_, between RRM and ssDNA, anisotropy data were fitted to eqs 1 and 2 and fitted functions were shown as solid curves. More than three independent experiments were performed to estimate error bars (standard deviations).

To get more insight into the distinct roles of RRM1 and RRM2 in the ssDNA/RNA binding of TDP-43, we have characterized the RRM-ssDNA interaction with fluorescence anisotropy. In this method, ssDNA modified with fluorescein at the 5′-end is titrated with an RRM protein, and the complex formation between RRM and ssDNA increases the anisotropy of fluorescence from fluorescein. In the pull-down assay, we used ssDNA with five repeats of TG and GG (Fig. 2), and similar results were actually obtained by reducing the repeat number from five to four (data not shown); therefore, ssDNAs containing four repeats of TG and GG ((TG)_4_ and A_3_(GG)_4_A_3_) were used for fluorescence anisotropy experiments. As shown in Fig. 3A, titrations of RRM1 to ssDNAs containing TG-repeated and GG-repeated sequences were found to increase fluorescence anisotropy, while no interaction was observed between RRM1 and AC-repeated ssDNA. We also confirmed the complex formation of RRM1 with UG-repeated ssDNA, in which thymine of (TG)_4_ was replaced with uracil, a substitute base in RNA (Supplemental Fig. S1A). It is also notable that RRM1 exhibited higher affinity toward (TG)_4_ (*K*_d_, 52 ± 12 nM) and (UG)_4_ (*K*_d_, 61 ± 30 nM) compared to A_3_(GG)_4_A_3_ (*K*_d_, 710 ± 200 nM); therefore, RRM1 contributes to the preferential binding of TDP-43 with TG/UG-repeated sequences. As mentioned above, RRM1 has been reported to accommodate nine nucleotides upon interaction with ssDNA/RNA^18^, but our results showed that four repeats of TG/UG still preserved the tight binding with RRM1 (and also RRM1–2, *vide infra*), and the observed binding affinity toward TG/UG-repeats was considerably comparable to those published previously^18^,^20^. In contrast, no complex formation was observed in RRM2 with all four fluorescein-modified ssDNAs tested in this study (Fig. 3B, Supplemental Fig. S1B). These results on RRM2 are completely consistent with the previous report showing no binding of RRM2 with TG/UG-repeated ssDNA/RNA^14^ and are also consistent with our findings in the pull-down assay that RRM1 but not RRM2 has ssDNA binding ability with moderate sequence-specificity.

When linked with RRM2 in tandem as in native TDP-43, RRM1 maintained high affinity to (TG)_4_ (*K*_d_, 77 ± 4.2 nM) and (UG)_4_ (*K*_d_, 150 ± 23 nM) ssDNA and showed no binding with the AC-repeated sequence (Fig. 3C, Supplemental Fig. S1C). Again, the tight binding of RRM1–2 with TG/UG-repeats is consistent with previous reports^17^,^20^. Quite interestingly, however, RRM1–2 almost completely lost the ability to form a complex with A_3_(GG)_4_A_3_ (Fig. 3C). Again, this result supports our proposal that RRM2 plays a fine-tuning role in the sequence-specific recognition of ssDNA by TDP-43.

We also confirmed that the same sequence-specificity of RRMs was observed by using RNA, the canonical substrate of TDP-43, instead of ssDNA. Namely, based upon fluorescence anisotropy experiments, RRM1 was found to bind fluorescein-modified (UG)_4_ and A_3_(GG)_4_A_3_ RNAs with 28 ± 9.0 and 390 ± 190 nM of *K*_d_, respectively, while RRM2 did not bind to either RNAs (Supplemental Fig. S2A and B). Furthermore, RRM1–2 was able to bind (UG)_4_ RNA with 3.5 ± 2.3 nM of *K*_d_ but lost its binding affinity to A_3_(GG)_4_A_3_ RNA (Supplemental Fig. S2C). Given a previous report showing that both RRM1 and RRM2 of mouse TDP-43 adopt a tetrameric state^19^, we thus suspect that the intricate interaction between the two RRMs might be a key to understanding the sequence-specific interaction with ssDNA/RNA.

### Forced RRM1/RRM2 interaction via a linker in tandem is required for the sequence-specific recognition of ssDNA

To reveal the effects of the RRM1/RRM2 interaction on the recognition of ssDNA, we first compared the ssDNA binding ability of RRM1 in the presence and absence of RRM2. Somewhat unexpectedly, RRM1 was found to bind both TG- and GG-repeated ssDNAs in a 1:1 mixture with RRM2 (Fig. 3D), implying no obvious role of RRM2 in the recognition of ssDNA. Moreover, we have tested the ssDNA binding ability of RRM2-1, in which the C-terminus of RRM2 (Glu186 - Asn267) is fused with the N-terminus of RRM1 (Val96 - Asp185), by the pull-down assay. The ten sequences of ssDNA described above were examined for their binding to RRM2-1, and we found that significant amounts of nucleotides were eluted with the wash step using TN-high buffer (Fig. 2A) in all of the sequences tested, suggesting no sequence-specificity of RRM2-1 in the binding of ssDNA (Supplemental Fig. S3). These results appear to contradict with our proposal that RRM2 increases the specificity in the sequence of ssDNA recognized by TDP-43; however, we later found that RRM2 would exert its fine-tuning function only when fused downstream of RRM1 in tandem.

**Figure 4:**
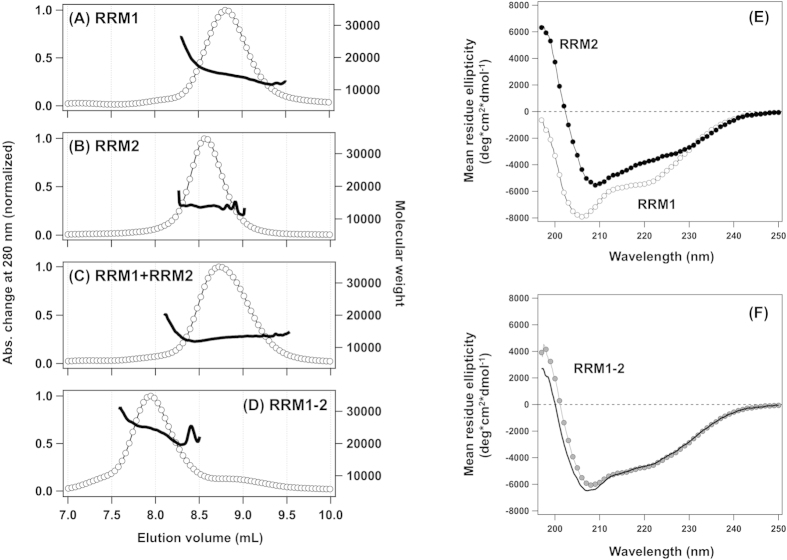
Limited interactions between RRM1 and RRM2 in their free forms. **(A)** 1 g/L (90 μM) RRM1, **(B)** 1 g/L (~100 μM) RRM2, **(C)** a mixture of 1 g/L RRM1 and 1 g/L RRM2, and **(D)** 1 g/L (~50 μM) RRM1–2 were analyzed by SEC-MALS. Chromatograms obtained by monitoring changes in absorbance at 280 nm are shown (open circles, left axis). Molecular weight of a species eluted from a gel filtration column was also analyzed with an on-line MALS and is shown in the chromatograms (a thick curve, right axis). **(E,F)** Secondary structures of RRM proteins were analyzed by CD spectroscopy: 20 μM RRM1 (open circles) and 20 μM RRM2 (filled circles) in (**E**), and 20 μM RRM1–2 (filled gray circles) in (**F**). A calculated CD spectrum of RRM1–2 was obtained by the sum of the CD spectra between RRM1 and RRM2 and is shown as a solid curve in (**F**).

Indeed, unlike mouse TDP-43, our gel filtration experiments monitored by multiple-angle light scattering have shown that both RRM1 (calc. MW: 12 kDa) and RRM2 (calc. MW: 11 kDa) from human TDP-43 favor a monomeric state (Fig. 4A,B), and no heterocomplex formation was observed when RRM1 was simply mixed with RRM2 (Fig. 4C). A tandem fusion of RRM1 and RRM2 (*i.e.* RRM1–2) was confirmed to elute earlier as a monomer (Fig. 4D, calc. MW: 22 kDa). We also considered structural changes on RRM1 and/or RRM2 by their linkage in tandem; however, the CD spectrum of RRM1–2 was almost fully described by the sum of spectra of RRM1 and RRM2 (Fig. 4E,F). Accordingly, while no major structural changes occur in the individual RRMs by their fusion in tandem, the physical connection of RRM1 followed by RRM2 in tandem as in native TDP-43 would force the RRMs to be arranged in a fixed orientation(s) and thereby exert the ability to bind ssDNA/RNA with a specific sequence.

### The fine-tuning role of RRM2 is compromised with mutational disruption of the forced interaction with RRM1 in RRM1–2

Genetic mutations in TDP-43 have been known as a cause of amyotrophic lateral sclerosis, and most of such pathogenic mutations are found in the C-terminal Gly-rich region of TDP-43 (Fig. 1A)^7^,^8^,^9^,^10^. Despite this, a pathogenic mutation, D169G, is located in RRM1 and occurs at the last β-strand in RRM1^8^, which is followed by a linker peptide to RRM2. Asp169 is not involved in the conventional binding site for (T/U)G-rich sequences^17^,^18^ but might either affect an alternative site or influence the relative orientation between RRM1 and RRM2. We therefore tested if D169G changed the sequence-specificity in the substrate recognition of RRM1–2; however, RRM1–2 (D169G) was able to bind TG-repeated but not GG-repeated ssDNA, suggesting that high sequence-specificity was maintained in the substrate recognition of mutant RRM1–2 (Supplemental Fig. S4A). Also, another pathogenic mutation, P112H, has been recently reported in RRM1^23^; however, the mutation did not affect the sequence-specificity of RRM1–2 in the binding of ssDNA (Supplemental Fig. S4B).

Although pathogenic mutations in RRM1–2 appear not to affect the sequence-specificity in substrate recognition, we suppose that any perturbations to the forced interaction between RRM1 and RRM2 will impact the recognition of ssDNA/RNA. Actually, a structure of RRM1–2 complexed with RNA substrate describes the fixed orientation of RRM1 and RRM2, which appears to be stabilized by a salt bridge between Arg151 of RRM1 and Asp247 of RRM2^17^. Furthermore, R151A/D247A double mutations in RRM1–2 have been shown to greatly reduce the ability to bind RNA with the sequence, 5′-GUGUGAAUGAAU-3′^17^. To test if the interaction between RRM1 and RRM2 in RRM1–2 is required for the increased specificity of substrate recognition, therefore, we repeated our fluorescence anisotropy experiments using RRM1–2 proteins with R151A and D247A mutations.

**Figure 5:**
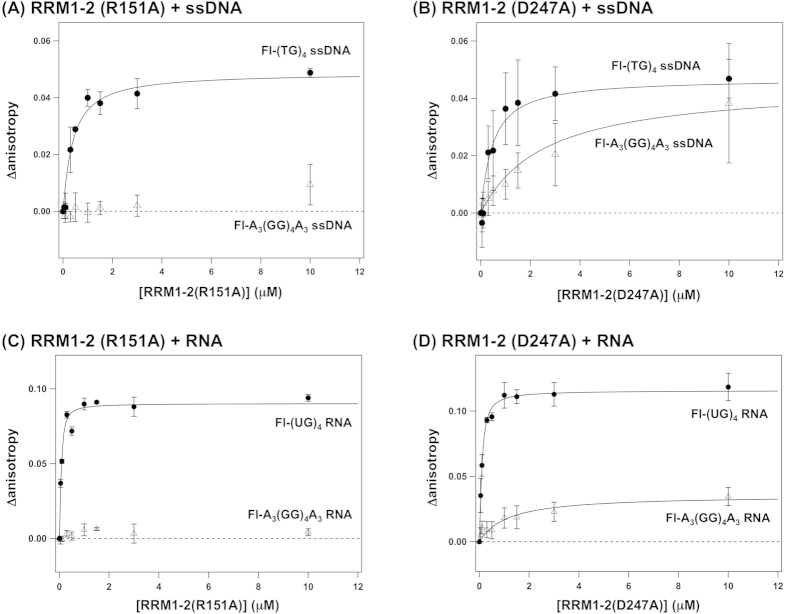
Sequence-specificity of RRM1–2 in the ssDNA recognition was significantly reduced by mutational perturbation on the interface between RRM1 and RRM2. Fluorescence anisotropy of (**A**,**B**) 0.1 μM Fl-(TG)_4_ (filled circles) and Fl-A_3_(GG)_4_A_3_ (open triangles) ssDNA and (**C,D**) 0.1 μM Fl-(UG)_4_ (filled circles) and Fl-A_3_(GG)_4_A_3_ (open triangles) RNA was measured in the presence of increasing amounts of RRM1–2 with (**A**,**C**) R151A and (**B,D**) D247A mutations. To estimate the dissociation constant, *K*_d_, between RRM and ssDNA/RNA, anisotropy data were fitted to eqs 1 and 2 and fitted functions were shown as solid curves. At least three independent experiments were performed to estimate error bars (standard deviations).

Quite notably, RRM1–2 with D247A was found to bind GG-repeated ssDNA as well as TG-repeats, while the binding of ssDNA with RRM1–2 was not significantly affected by R151A mutation (Fig. 5A,B). Similarly, UG-repeated RNA (Fl-(UG)_4_) was also recognized by both RRM1–2 constructs with R151A and D247A mutations (33.8 ± 12.6 nM and 54.6 ± 7.5 nM, respectively: Fig. 5C,D). This is not because the number of nucleotides in Fl-(UG)_4_ (*i.e.* eight) was less than that of the number of interaction sites in RRM1–2 (*i.e.* ten); indeed, we have confirmed that RRM1–2(R151A) and RRM1–2(D247A) tightly binds Fl-(UG)_12_ with 105 ± 32 nM and 122 ± 35 nM of *K*_d_, respectively (data not shown). In contrast, RRM1–2(D247A) but not RRM1–2(R151A) interacted with GG-repeated RNA (Fl-A_3_(GG)_4_A_3_) (Fig. 5C,D). Given the relatively small increase in anisotropy, the affinity of RRM1–2(D247A) with GG-repeated RNA may be weaker than that with GG-repeated ssDNA. Collectively, therefore, these results show that D247A but not R151A mutation decreases the sequence-specificity of RRM1–2 constructs toward the recognition of ssDNA and RNA.

**Figure 6:**
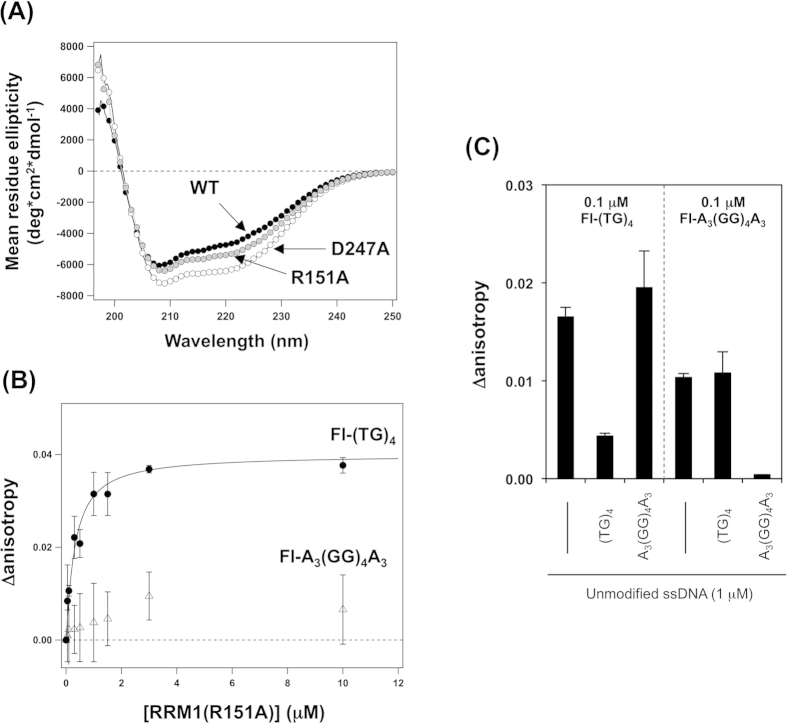
Distinct binding modes in RRM1 are involved in sequence-specific recognition of nucleic acids. (**A)** CD spectra of 20 μM RRM1–2 with R151A (gray filled circles) and D247A (open circles) were shown. For comparison, a CD spectrum of RRM1–2 in Fig. 4F was again shown as filled circles. **(B)** Fluorescence anisotropy of 0.1 μM Fl-(TG)_4_ (filled circles) and Fl-A_3_(GG)_4_A_3_ (open triangles) ssDNA was measured with increasing amounts of RRM1 with R151A mutation. Experimental procedures were the same with those in (Fig. 3A). **(C)** Competition experiments of ssDNA binding on RRM1 probed by fluorescence anisotropy. RRM1 (1 μM) was mixed with 0.1 μM of fluorescein-modified ssDNA (left, Fl-(TG)_4_; right, Fl-A_3_(GG)_4_A_3_) in a buffer containing 50 mM Tris and 100 mM NaCl at pH 8.0. Unmodified ssDNA (1 μM) was further added, and the changes in the fluorescence anisotropy were then monitored. The data were represented as Δanisotropy, in which the anisotropy value of fluorescein-modified ssDNA alone (0.1 μM) was subtracted from the observed ones.

We also confirmed minimal changes in CD spectra (Fig. 6A) of RRM1–2 with R151A and D247A mutations, implying no significant effects on the solution structure of RRM1–2. While oligomeric species were detected in RRM1–2 (D247A) as a minor population in gel filtration experiments (Supplemental Fig. S5), which is consistent with the stabilizing role of Asp247 in the conformation of RRM2^21^, loss of sequence-specificity by D247A mutation is consistent with our hypothesis that sequence-specific recognition of ssDNA/RNA is controlled by the forced interaction between RRM1 and RRM2 within RRM1–2. Little effect of the R151A mutation on the binding of ssDNA/RNA were, however, somewhat unexpected, because a salt bridge interaction between Arg151 and Asp247 would be disrupted in both R151A and D247A mutant RRM1–2 proteins.

In many DNA/RNA-binding proteins, an Arg residue supports the binding of DNA/RNA through electrostatic and/or hydrogen bonding interactions^15^. We thus expected that Arg151 in RRM1 could also act as a binding site for ssDNA/RNA^11^ but became no longer available in RRM1–2 due to the formation of a salt-bridge interaction with Asp247 in RRM2. In other words, Arg151 in RRM1 may be a part of the binding site specifically for GG-repeated nucleotides. To test this idea, we examined if RRM1 loses the ability to bind GG-repeated ssDNA by introduction of the R151A mutation. As expected, RRM1(R151A) was found to bind TG-repeated (*K*_d_, 210 nM) but not GG-repeated ssDNA (Fig. 6B). The availability of Arg151 would, therefore, determine the binding of GG-repeated but not TG-repeated ssDNA by RRM1. Namely, Arg151 in RRM1 is considered as a key player in sequence-specific recognition of ssDNA/RNA by TDP-43.

### A proposed mechanism regulating the sequence-specific recognition of TDP-43

TDP-43 regulates alternative splicing of the cystic fibrosis transmembrane regulator (CFTR) and facilitates the skipping of the CFTR exon 9^12^. A previous study has shown increased levels of exon 9 by R151A and D247A mutations in TDP-43, suggesting the loss of its functional activity^17^. Accordingly, specific salt-bridge interactions between RRM1 and RRM2 have been considered to play important roles in the TDP-43 splicing function. In contrast, we have shown here that apparent sequence-specificity for the substrate recognition of TDP-43 is maintained by R151A but not D247A mutations (Fig. 5). The exon-skipping activity of TDP-43 in the splicing of CFTR mRNA is hence not correlated with sequence-specificity regulated by the salt bridge interaction between Arg151 and Asp247. While other as-yet-unknown physiological function(s) of TDP-43 could be affected by such non-specific interactions (*e.g.* RRM1 and GG-repeats), we would like to emphasize here that several binding modes are possible between RRM1 and ssDNA/RNA in a sequence-dependent manner.

To strengthen this idea, we examined if TG and GG-repeated ssDNAs compete with each other to bind to RRM1 of TDP-43. As shown in Fig. 6C, 0.1 μM Fl-(TG)_4_ has increased fluorescence anisotropy upon addition of 1 μM RRM1, confirming the formation of RRM1/Fl-(TG)_4_ complex. As expected, upon further addition of 1 μM unmodified (TG)_4_, the anisotropy increase was almost cancelled out, consistent with the competition of Fl-(TG)_4_ and (TG)_4_ for the same binding site on RRM1. In contrast, anisotropy did not decrease when 1 μM unmodified A_3_(GG)_4_A_3_ was added to the mixture of 1 μM RRM1 and 0.1 μM Fl-(TG)_4_, (Fig. 6C). This might be because the affinity of RRM1 for GG-repeated ssDNA was weaker than that for TG-repeats (Fig. 6C). Nonetheless, the concentration of A_3_(GG)_4_A_3_ (1 μM) was 10-fold higher than that of Fl-(TG)_4_ (0.1 μM), and the anisotropy would thus be decreased if those two ssDNAs competed for the same binding site. These results imply that Fl-(TG)_4_ and A_3_(GG)_4_A_3_ bind at separate distinct sites on RRM1.

To further test if TG and GG-repeated ssDNAs bind to distinct sites on RRM1, we repeated the competition experiments using Fl-A_3_(GG)_4_A_3_. As expected, the fluorescence anisotropy of 0.1 μM Fl-A_3_(GG)_4_A_3_ increased upon addition of 1 μM RRM1, and the increase was almost cancelled out when 1 μM unmodified A_3_(GG)_4_A_3_ was further added (Fig. 6C). These results have shown that Fl-A_3_(GG)_4_A_3_ and A_3_(GG)_4_A_3_ competes for the same binding site on RRM1. Quite notably, the anisotropy increase of 0.1 μM Fl-A_3_(GG)_4_A_3_ upon addition of 1 μM RRM1 was not affected by addition of 1 μM unmodified (TG)_4_ (Fig. 6C). Compared to A_3_(GG)_4_A_3_, (TG)_4_ had 10-fold higher affinity to RRM1 and were present in 10-fold excess in this experimental condition. Despite this, little effects of (TG)_4_ on the anisotropy increase by the complexation between RRM1 and Fl-A_3_(GG)_4_A_3_ were observed. Collectively, therefore, these competition experiments support and strengthen our idea that RRM1 has several distinct binding sites at least for TG and GG-repeated ssDNA.

**Figure 7:**
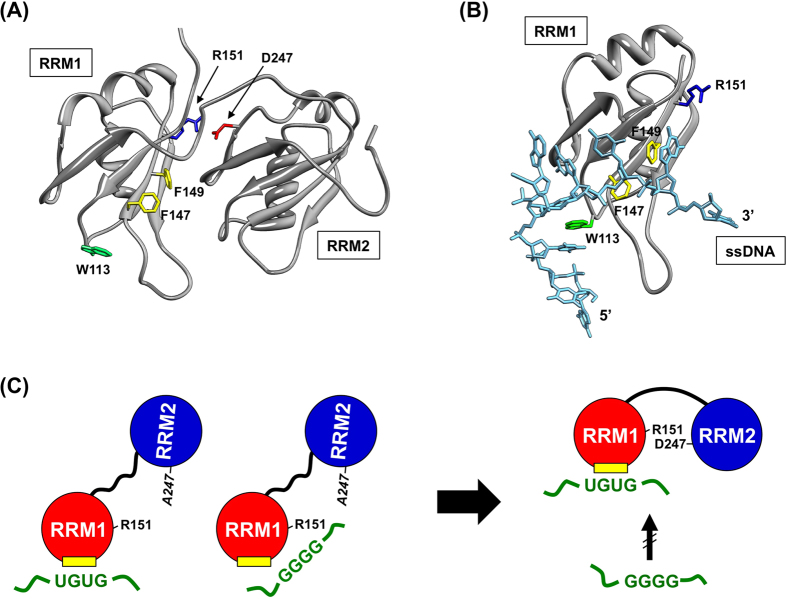
A proposed mechanism regulating the sequence-specific recognition of RNA/ssDNA by TDP-43 **(A)** A three-dimensional structure of RRM1–2 (PDB ID: 4BS2). Arg151 (blue) in RRM1 forms salt bridge interactions with Asp247 (red) in RRM2. Trp113 (green) and Phe147/149 (yellow) have been proposed as a binding site in RRM1 for ssDNA/RNA. **(B)** A three-dimensional structure of RRM1 complexed with ssDNA rich in TG (colored cyan) (PDB ID: 4IUF). Arg151 (blue), Trp113 (green), and Phe147/149 (yellow) are also indicated. **(C)** A “canonical” binding site in RRM1 for TG/UG-repeats is colored yellow, and its recognition by RRM1–2 does not require Arg151. RRM1 has an alternative binding site composed of Arg151 for ssDNA/RNA other than TG/UG-repeats, although canonical and alternative binding sites are partially overlapped. Disrupted interactions between RRM1 and RRM2, for example, by mutation at Asp247 to Ala would make Arg151 in RRM1 available for binding GG-repeated ssDNA/RNA (left). In contrast, Arg151 in wild-type RRM1–2 has salt bridge interactions with Asp247, and RRM2 is considered to occlude the alternative binding site for GG-repeats but not the canonical binding site for TG/UG-repeats (right). The availability of Arg151 in RRM1 thus affects the sequence-specific binding of ssDNA by TDP-43.

Nonetheless, we found that tandem fusion of RRM1 with RRM2 resulted in the predominant preference to TG/UG-repeated ssDNA/RNA. While the isolated RRM1 and RRM2 did not interact with each other, the tandem fusion of those two RRMs as in native TDP-43 is considered to stabilize their interactions in a fixed orientation(s)^17^. Actually, such fixed orientation of RRM1 and RRM2 has been shown to add two nucleotide-specific interactions in RRM2 to the recognition surface on TDP-43^17^. As described above, however, our experimental results suggest two distinct sites for the binding of nucleotides in RRM1. More specifically, Arg151 in RRM1 would have roles in the binding of nucleotides containing GG-repeats but is not involved in the recognition surface for TG/UG-rich nucleotides reported so far^18^. Furthermore, the linker connecting RRM1 and RRM2 appears to spatially separate Arg151 from an ssDNA/RNA binding site composed of Trp113 and Phe147/149 (Fig. 7A). Accordingly, tandem-fused RRM2 with a linker not only provides nucleotide-specific interactions but probably also occludes the alternative ssDNA/RNA binding site(s) in RRM1 involving Arg151, which eventually increases the sequence-specificity of ssDNA recognized by TDP-43.

In the isolated RRM1, Arg151 is exposed to the solvent and would be available for the binding of ssDNA. As mentioned above, however, a crystal structure of RRM1 complexed with a TG-rich ssDNA shows that Arg151 is not involved in the interaction with ssDNA (Fig. 7B)^18^. This is consistent with our findings that RRM1 with R151A mutation can bind TG-repeats (Fig. 6B). In the direction from the 5′-end to the 3′-end of the bound ssDNA, π-π stacking interactions with Trp113 and Phe149 are formed, but the DNA strand then bends away from RRM1, resulting in little contribution of Arg151 to the binding of the TG-rich ssDNA (Fig. 7B)^18^. Given that Arg151 could interact with the 3′-region of the substrate ssDNA, repetitive adenines in the 3′-end of our GG-repeated ssDNA (5′-A_3_(GG)_4_A_3_-3′) may be important in the interaction with Arg151. Also notably, the interactions of RRM1 with TG-repeats and GG-repeats were similarly retarded by substitution of Trp113 with Ala (W113A) (Fig. 7B; Supplemental Fig. S6), suggesting that the binding site(s) in RRM1 for TG- and GG-repeats partially overlaps. While it remains unclear how our GG-repeated ssDNA/RNA is bound to RRM1, the distinct conformation of ssDNA/RNA that is dependent upon its sequence would allow for the interaction with RRM1 through Arg151. Also, the N-terminal domain of TDP-43 (1 – 105, also see Fig. 1A) has been shown to increase the affinity of RRM1–2 toward TG-repeated ssDNA^24^ and was recently found to directly bind TG-repeated ssDNA^25^. Each of the domains in TDP-43 would, therefore, play a distinct role in recognizing the substrate nucleic acids in a sequence-specific manner, and RRM2 is considered to act as a steric switch to regulate the sequence-specificity in the substrate binding of TDP-43 (Fig. 7C).

Cytoplasmic mislocalization of nuclear TDP-43 has been characterized as a pathological hallmark of ALS diseases and proposed to associate with protein misfolding/aggregation. The conformational information of misfolded TDP-43 is, however, quite limited^20^,^26^,^27^. In this study, we could not find obvious effects of pathogenic mutations in RRM1 (P112H, D169G) on the sequence-specificity in the substrate binding. Also, pathogenic mutations in TDP-43 are concentrated in the C-terminal auxiliary region that follows RRM1–2^7^,^8^,^9^,^10^, and it remains unknown whether the C-terminal mutations affect the conformation of RRM1–2 and how. Quite notably, in the affected ALS patients, pathological TDP-43 abnormally localized at the cytoplasm was immunostained by a monoclonal antibody recognizing a conformational epitope at Asp247; in contrast, normal nuclear TDP-43 in the non-ALS control subjects was not detected^21^. Asp247 is thus considered to become exposed in misfolded TDP-43 at the cytoplasm, by which the salt-bridge interaction between Arg151 and Asp247 would be lost. Also interestingly, Asp247 is a part of the nuclear exporting signal (NES) of TDP-43^28^ but appears to be buried in the interface between RRM1 and RRM2^17^. Weakening the inter-subunit interactions between RRM1 and RRM2 may thus facilitate the cytoplasmic mislocalization of TDP-43 by exposing the NES including Asp247 and also disturb the RNA metabolism by compromising the sequence-specificity in the substrate binding of TDP-43 due to the exposure of Arg151.

## Methods

### Preparation of RRM proteins

For preparation of recombinant RRM proteins, cDNAs corresponding to RRM1 (Val96 - Asp185), RRM2 (Glu186 - Asn267), and RRM1–2 (Val96 - Asn267) were inserted between CAT and ATG codons of the NdeI site in a plasmid vector, pET15b (Novagen). Furthermore, a peptide linker composed of GSSGSSG was inserted just after the CAT codon. RRM2 has no Trp residue, which makes it difficult to determine the concentration spectroscopically; therefore, in RRM2, a TGG codon that codes Trp was also introduced after the CAT codon. Point mutations were introduced by the inverse PCR method using a KOD-FX-neo DNA polymerase (TOYOBO). All constructs in this study were confirmed by DNA sequencing.

*E. coli* BL21(DE3) (New England Biolabs) was transformed with the respective plasmids, and expression of RRM proteins was induced with 0.5 mM isopropyl-1-thio-β-D-galactopyranoside at 37 °C for 6 hours (RRM1 and RRM2) or at 20 °C for 20 hours (RRM1–2) in LB medium with ampicillin. RRM1 with R151A mutation was co-expressed with GroEL and GroES (pGro7, TAKARA) for assisting the folding of the soluble protein. RRM proteins were then purified with Profinity IMAC Ni-Charged Resin (Bio-Rad); after loaded with cleared lysates, the resins were washed with 50 mM Tris/1 M NaCl, pH 8.0 (TN-high buffer) and then with 50 mM Tris/100 mM NaCl, pH 8.0 (TN-low buffer) containing 10 mM imidazole. RRM proteins expressed from our constructs have a sequence recognized by a specific protease, thrombin, between an N-terminal His tag and the GSSGSSG linker; therefore, RRM proteins were eluted from the resins by incubation with thrombin (GE Healthcare) at 4 °C for 24 hours. A successful purification of RRM proteins including the removal of an N-terminal His-tag was confirmed by SDS-PAGE. The concentration of RRM proteins was spectroscopically determined from the absorbance at 280 nm in the presence of 6 M guanidine hydrochloride.

### Pull-down assay for ssDNA binding of RRM proteins

As mentioned, the cleared lysates were mixed with Ni^2+^-affinity resins and washed with a TN-high buffer followed by a TN-low buffer containing 10 mM imidazole. The resins were equally aliquoted into 1.5-mL tubes, to one of which a TN-low buffer containing 250 mM imidazole was added for elution of RRM proteins; thereby, the RRM proteins bound to the resins could be quantified spectroscopically (~3 nmol in RRM1 and RRM2; ~2 nmol in RRM1–2). Then, 0.1 nmol of ssDNA was added to the other tubes, which were incubated at room temperature for an hour. After washing with a TN-low buffer containing 10 mM imidazole, the resins were incubated in a TN-high buffer for dissociating the bound ssDNA from RRM proteins on the resins, and the eluate was collected as the ssDNA fraction. A TN-low buffer with 250 mM imidazole was further added to the resins, by which the RRM proteins were eluted and collected as the RRM fraction. UV/vis absorption spectra of both the ssDNA fraction and the RRM fraction was measured, and the ssDNA bound to the RRM proteins was quantified from the absorption at 260 nm.

### Fluorescence anisotropy measurements

Fluorescence anisotropy was measured using a fluorescence spectrophotometer (F-4500, Hitachi) equipped with a polarizer system. Sequences of ssDNA examined were (TG)_4_, (UG)_4_, (AC)_4_, and A_3_(GG)_4_A_3_, each of which was covalently modified with fluorescein at its 5′-end (Eurofins Genomics). We have also prepared fluorescein-modified (GG)_4_; however, compared to the other sequences, much weaker intensity of fluorescence was found, which is possibly due to efficient energy transfer between fluorescein and guanine. For GG-repeats, therefore, we used A_3_(GG)_4_A_3_, in which comparable intensity of fluorescence was observed by keeping fluorescein away from guanine via three adenines.

Fluorescein-modified ssDNA (0.1 μM) was prepared in a TN-low buffer with RRM proteins, the final concentration of which was in the range from 0 to 30 μM. After a sample in a quartz cuvette was left at 10 °C for 5 min., the fluorescence anisotropy was measured with 488 and 516 nm of excitation and emission wavelength, respectively. The dissociation constant, *K*_d_, between RRM proteins and ssDNA was estimated by fitting the observed anisotropy (*r*) with the following equations;


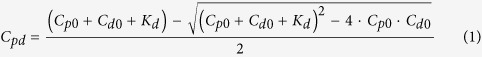



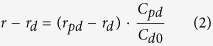


where *C*_p0_, *C*_d0_, and *C*_pd_ are concentrations of total RRM, total ssDNA, and RRM-ssDNA complex, respectively. Also, *r*_d_ and *r*_pd_ are the anisotropy values of ssDNA alone and RRM-ssDNA, respectively.

We have also prepared (UG)_4_ and A_3_(GG)_4_A_3_ RNAs covalently modified with fluorescein at 5′-end (FASMAC) and examined the interactions between the RRMs and those RNAs by fluorescence anisotropy experiments. Experimental conditions for RNA were the same with those for ssDNA as described above, but RNase inhibitor, RNasin (Promega), was further added in the sample solution for preventing adventitious degradation of RNA.

### Circular dichroism (CD) spectroscopy

For the measurement of far-UV CD spectra (J-720WI, Jasco), 20 μM RRM proteins were prepared in a buffer containing 10 mM Na-Pi and 100 mM NaCl at pH 7.0.

### Size exclusion chromatography with an on-line multi-angle light scattering (SEC-MALS)

RRM proteins (2, 1, and 0.5 g/L) in 10 mM Na-Pi/100 mM NaCl, pH 7.0 were loaded onto a gel filtration column (TSKgel G2000SW, TOSOH) fitted to the HPLC system (Shimadzu), and the absorbance changes at 280 nm was monitored. A molecular size of the protein eluted from the column was also determined by multi-angle light scattering using miniDAWN TREOS (WYATT Technology) connected on-line to the HPLC system.

## Additional Information

**How to cite this article**: Furukawa, Y. *et al*. A molecular mechanism realizing sequence-specific recognition of nucleic acids by TDP-43. *Sci. Rep.* **6**, 20576; doi: 10.1038/srep20576 (2016).

## Supplementary Material

Supplementary Information
