# Supplementary material for: A molecular mechanism realizing sequence-specific recognition of nucleic acids by TDP-43
Source: Sci Rep. 2016 Feb 3;6:20576. doi: 10.1038/srep20576 (PMC4738331; doi:10.1038/srep20576)
Supplement: Supplementary Information [file srep20576-s1.pdf]

SUPPLEMENTAL MATERIALS

A molecular mechanism realizing sequence-specific recognition of nucleic acids by TDP-43

Yoshiaki Furukawa[1,*], Yoh Suzuki[1], Mami Fukuoka[1], Kenichi Nagasawa[1], Kenta Nakagome[1], Hideaki Shimizu[2], Atsushi Mukaiyama[3,4] and Shuji Akiyama[3,4]

[1]From Laboratory for Mechanistic Chemistry of Biomolecules, Department of Chemistry, Keio University, Yokohama 223-8522, JAPAN
[2] RIKEN Center for Life Science Technologies, Yokohama 230-0045, JAPAN
[3] Research Center of Integrative Molecular Systems (CIMoS), Institute for Molecular Science, Okazaki 444-8585, JAPAN
[4] Department of Functional Molecular Science, SOKENDAI (The Graduate University for Advanced Studies), Okazaki 444-8585, JAPAN.

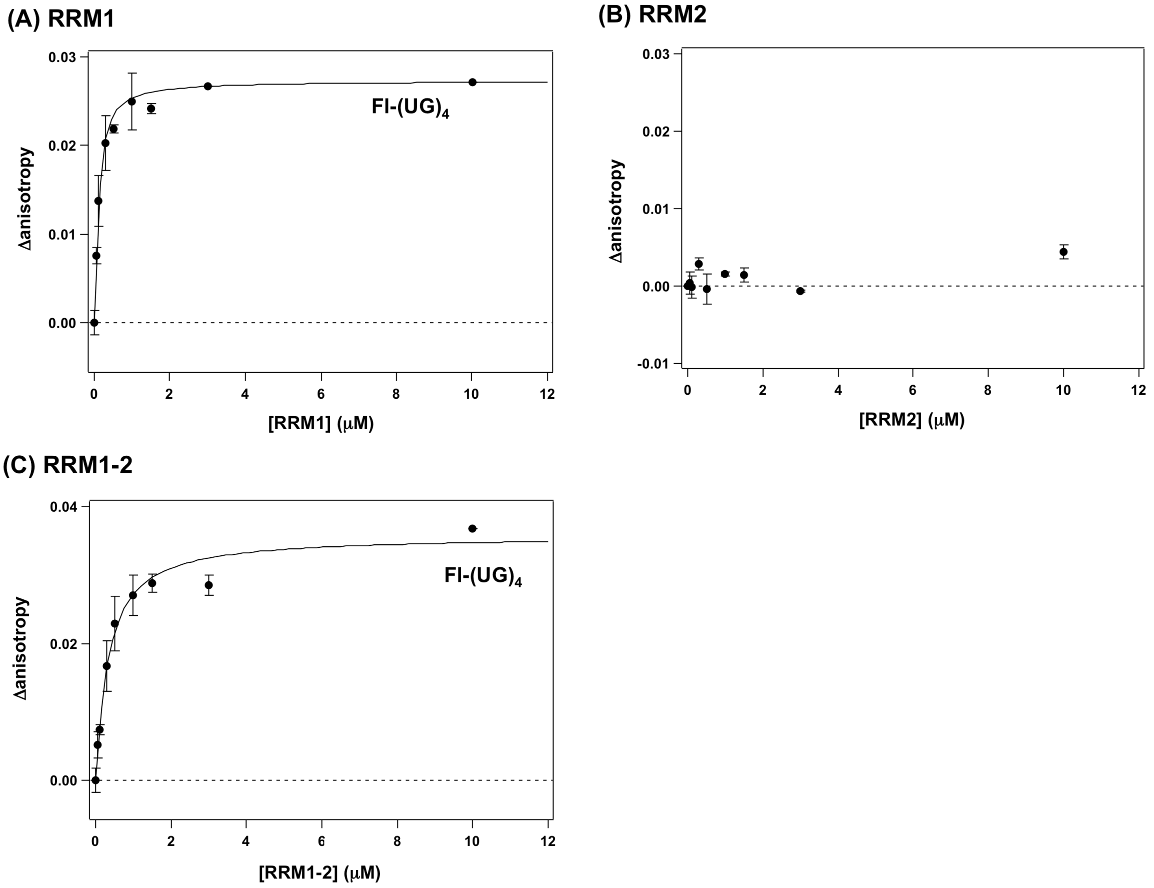

Figure S1 Analysis of the interaction between RRM proteins and fluorescently labeled $(UG)_4$ ssDNA by fluorescence anisotropy measurements. 0.1 μM Fl-$(UG)_4$ was titrated with **(A)** RRM1, **(B)** RRM2, and **(C)** RRM1-2, and the fluorescence anisotropy was measured. More than three independent experiments were performed to estimate error bars (standard deviations).

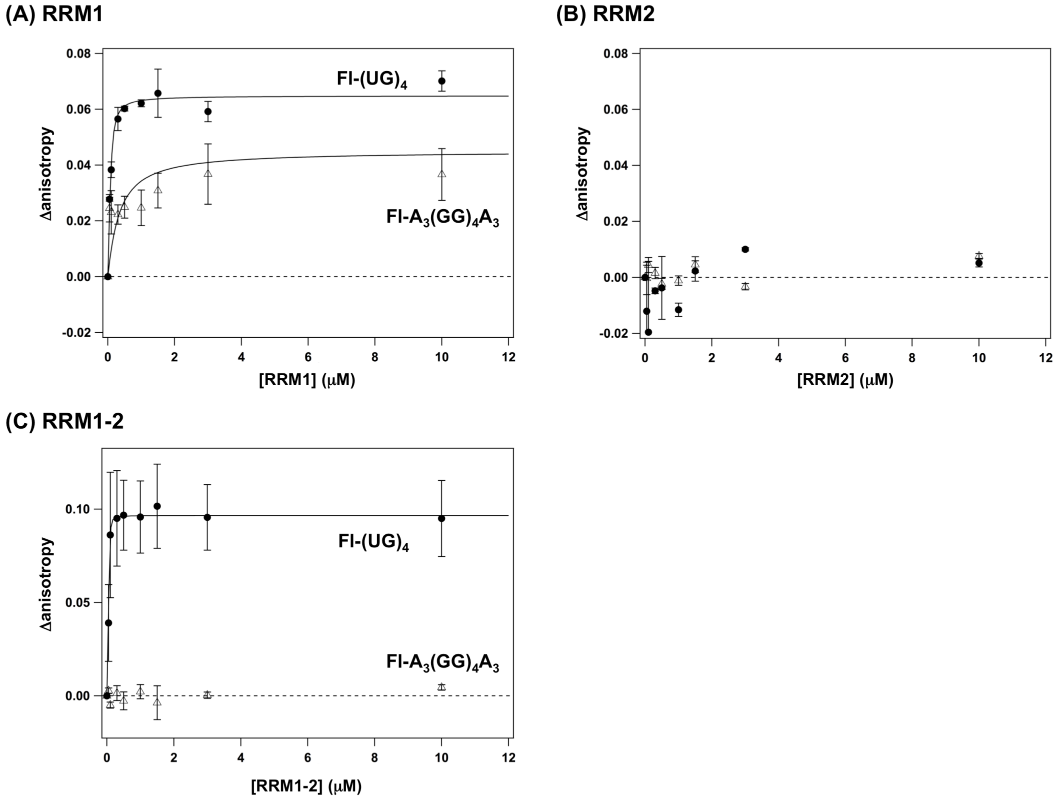

Figure S2 Analysis of the interaction between RRM proteins and fluorescently labeled RNA by fluorescence anisotropy measurements. 0.1 μM Fl-(UG)$_4$ or Fl-A$_3$(GG)$_4$-A$_3$ RNA was titrated with **(A)** RRM1, **(B)** RRM2, and **(C)** RRM1-2, and the fluorescence anisotropy was measured. More than three independent experiments were performed to estimate error bars (standard deviations).

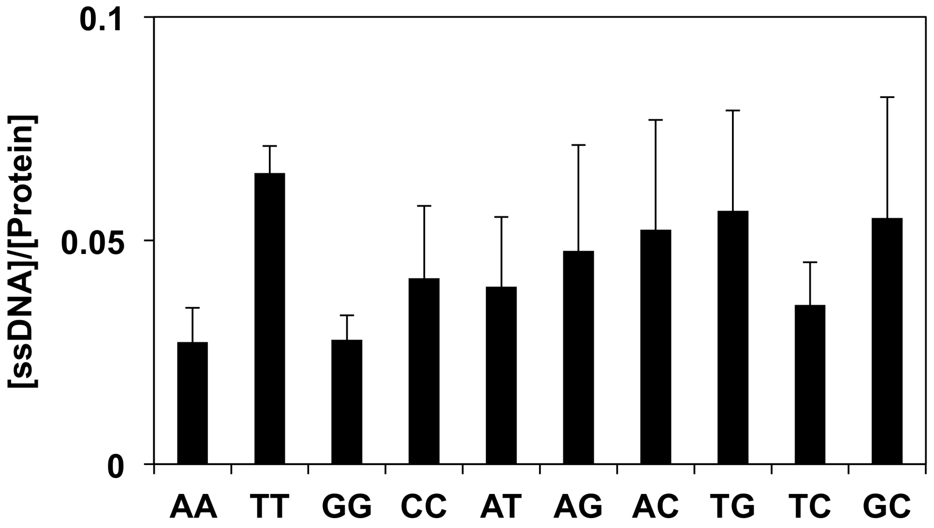

Figure S3 No sequence-specificity of RRM2-1 for the binding of ssDNA. The molar ratio between ssDNA and RRM2-1 in a pull-down assay was plotted against NN', which describes a repeating part in an ssDNA sequence, 5'-$A_{10}(NN')_5A_{10}$-3'. An exception is TT, which is an ssDNA with the sequence, 5'-$C_{10}(TT)_5C_{10}$-3'. Three independent experiments were performed to estimate error bars (standard deviations).

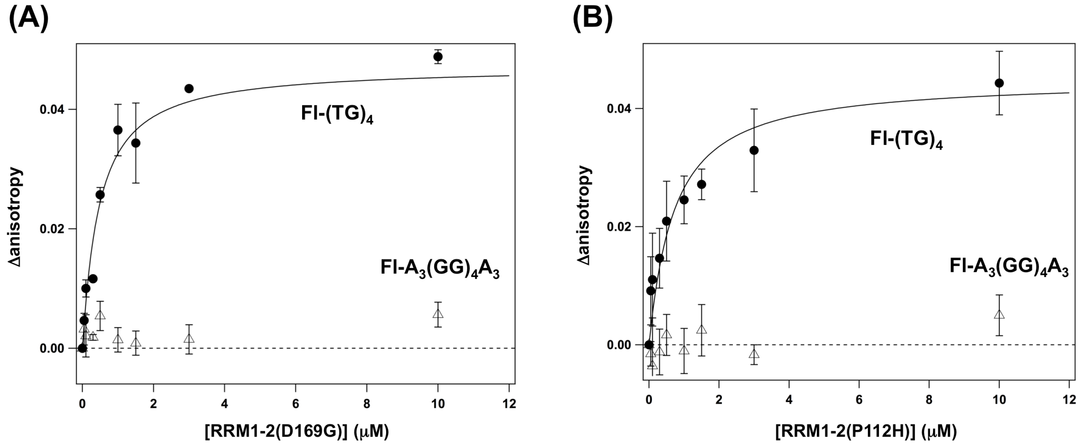

Figure S4 Analysis of the interaction between RRM1-2 with pathogenic mutations and fluorescently labeled ssDNA by fluorescence anisotropy measurements. 0.1 μM Fl-(UG)$_4$ (filled circles) and Fl-A$_3$(GG)$_4$A$_3$ (open triangles) were titrated with RRM1-2 with **(A)** D169G and **(B)** P112H mutations, and the fluorescence anisotropy was measured. More than three independent experiments were performed to estimate error bars (standard deviations).

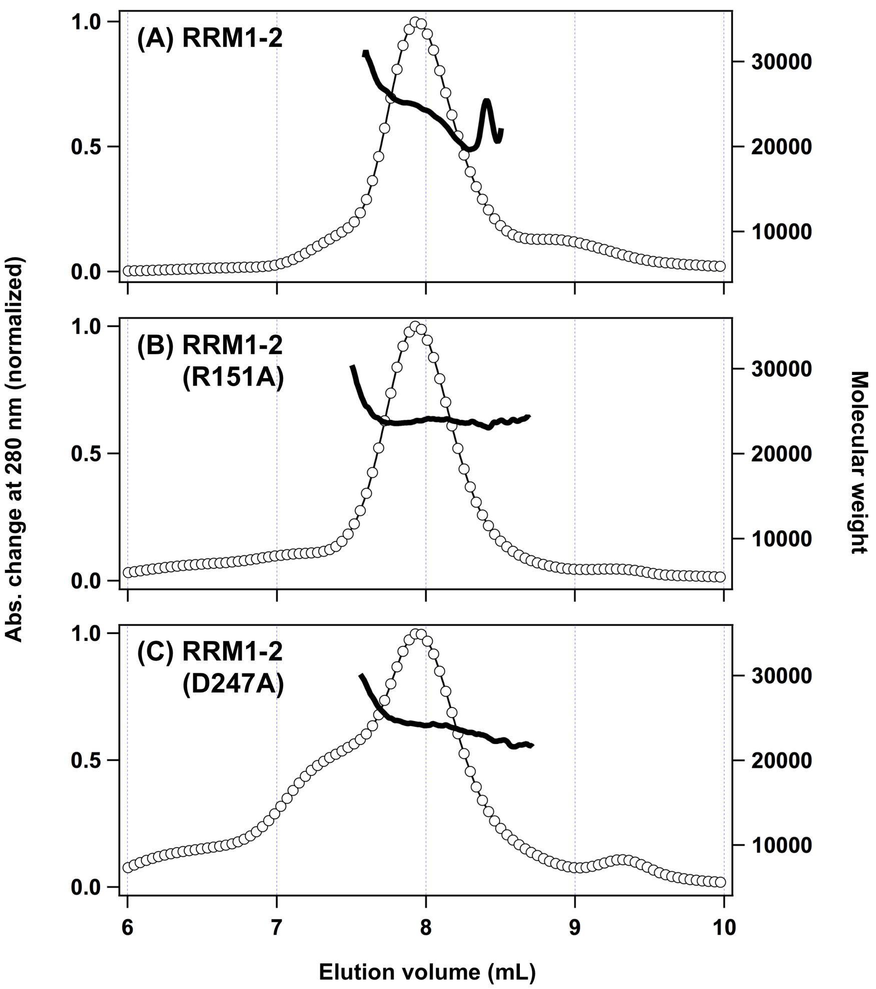

Figure S5 A quaternary structure of RRM1-2 was analyzed by size-exclusion chromatography. **(A)** RRM1-2, **(B)** RRM1-2 with R151A mutation, and **(C)** RRM1-2 with D247A mutation in 1 g/L (50 μM) concentration were analyzed by SEC-MALS. Chromatograms obtained by monitoring changes in absorbance at 280 nm were shown (open circles, left axis). Molecular weight of a species eluted from a gel filtration column was also analyzed with an on-line MALS and shown in the chromatograms (a thick curve, right axis).

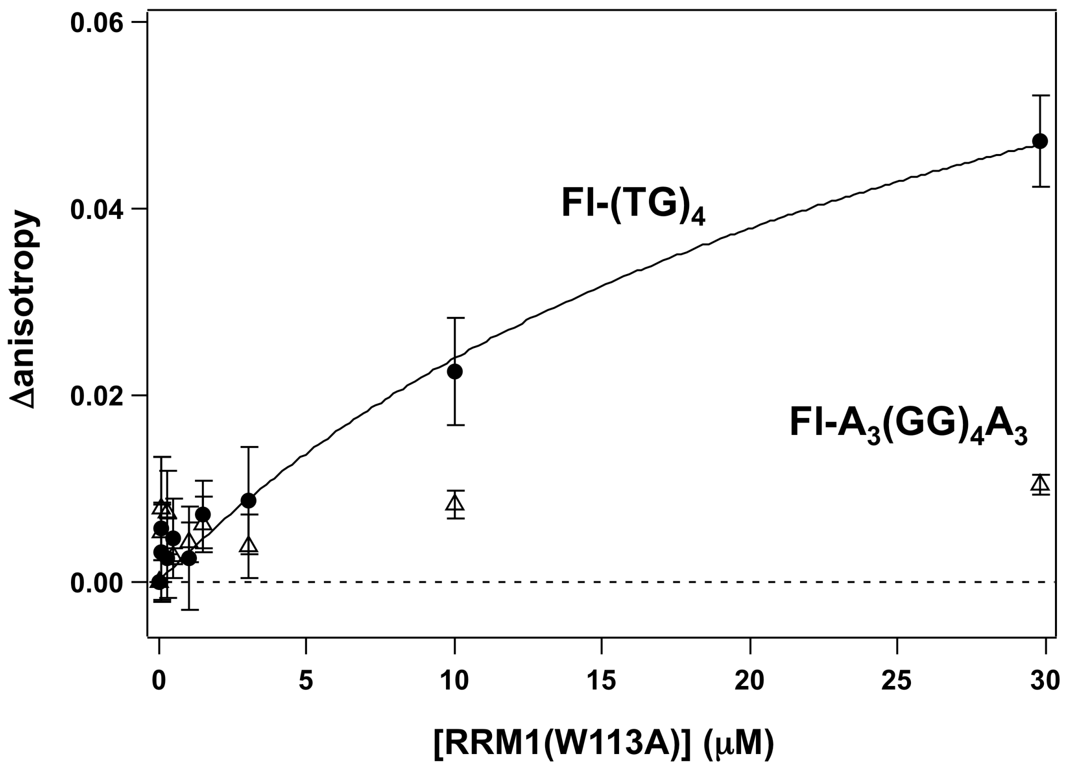

Figure S6 Analysis of the interaction between RRM1 with W113A mutation and fluorescently labeled ssDNA by fluorescence anisotropy measurements. 0.1 μM Fl-(UG)$_4$ (filled circles) and Fl-A$_3$(GG)$_4$A$_3$ (open triangles) were titrated with RRM1 with W113A mutation, and the fluorescence anisotropy was measured. More than three independent experiments were performed to estimate error bars (standard deviations).
